# Transfusion Support in Hematopoietic Stem Cell Transplantation: A Contemporary Narrative Review

**DOI:** 10.46989/001c.94135

**Published:** 2024-03-25

**Authors:** Brian D. Adkins, Jeremy W. Jacobs, Garrett S. Booth, Bipin N. Savani, Laura D. Stephens

**Affiliations:** 1 Pathology The University of Texas Southwestern Medical Center; 2 Laboratory Medicine and Pathology Mayo Clinic; 3 Pathology, Microbiology, and Immunology Vanderbilt University Medical Center; 4 Internal Medicine, Division of Hematology/ Oncology Vanderbilt University Medical Center; 5 Pathology University of California, San Diego

**Keywords:** transfusion support, hematopoietic stem cell tranplant, HLA, alloimmunization, transfusion thresholds

## Abstract

Hematopoietic stem cell transplantation (HSCT) is a cornerstone of modern medical practice, and can only be performed safely and effectively with appropriate transfusion medicine support. Patients undergoing HSCT often develop therapy-related cytopenia, necessitating differing blood product requirements in the pre-, peri-, and post-transplant periods. Moreover, ensuring optimal management for patients alloimmunized to human leukocyte antigens (HLA) and/or red blood cell (RBC) antigens, as well as for patients receiving ABO-incompatible transplants, requires close collaboration with transfusion medicine and blood bank professionals. Finally, as updated transfusion guidelines and novel blood product modifications emerge, the options available to the transplant practitioner continue to expand. Herein, we detail contemporary blood transfusion and transfusion medicine practices for patients undergoing HSCT.

## Introduction

Blood transfusion support for patients undergoing hematopoietic stem cell transplantation (HSCT) is an essential component of their care. The ability to provide safe and effective blood products throughout the pre-, intra-, and post-transplantation periods is critical for optimizing overall survival and outcomes in this population. To that end, there are specific considerations both transplant and blood banking services must take into account for these patients. Moreover, as the number of indications for HSCT continues to expand, and as cellular therapy treatment modalities evolve, these principles will continue to develop and remain relevant for the foreseeable future.

The recent increase in the number and type of diseases for which HSCT may be attempted, coupled with the rise of cord blood utilization, haploidentical transplant, and permissive human leukocyte antigen (HLA) mismatching, has contemporaneously coincided with emerging evidence-based use of restrictive red blood cell (RBC) and platelet (PLT) transfusions.^1–5^ Accumulating experience has also led to better characterization of pediatric HSCT, and HSCT in non-malignant conditions such as sickle cell disease.^6,7^ While an exhaustive, detailed review of every disease process managed with HSCT is beyond the scope of this article, important pearls will be highlighted. Herein we will describe general transfusion support for patients undergoing HSCT.

## General Transfusion Considerations

Although thresholds for blood transfusion in patients receiving HSCT are similar to those for patients with cytopenias, the anticipated progression from pre-transplantation through preparative regimen and eventual engraftment allows the clinician to anticipate patient needs. Patients with suboptimal pre-transplant hematopoietic cell production will require more transfusions throughout the induction and engraftment period, and pre-HSCT thrombocytopenia or RBC transfusion predict this increased need.^8–10^ To that end, the same studies have shown that a lower CD34-positive cell yield is predictive of increased transfusion burden post HSCT.^8,9^ Finally, patients undergoing haploidentical HSCT or HSCT from an umbilical cord graft will have increased transfusion burden during the engraftment period.^1^ In practice, the majority of patients undergoing autologous or allogeneic HSCT will receive at least one cellular blood product during the transplant process, with most patients (64-88%) receiving RBC transfusion and nearly all (97-100%) receiving PLT transfusion.^1,8^

## Pretransfusion Testing

ABO and RhD typing must be performed on both allogeneic donors and recipients prior to transplantation.^11^ An antibody detection test shall be performed on allogeneic recipients and may also be considered in donors.^11^ For patients with diseases that predispose to alloimmunization (e.g., myelodysplastic syndrome), the transplant center may consider RBC antigen typing and providing RBCs matched for the RhD, RhCE, and K (Kell) antigens, to prevent alloantibody formation, though this is not feasible at all sites.^12,13^ Some patients undergoing HSCT may have received prior monoclonal antibody therapy that can interfere with pretransfusion testing. Knowledge of the patient’s medication history is key to recognizing and potentially mitigating drug-interference (e.g., anti-CD38 and anti-CD47 monoclonal antibody therapies).

Identification of an RBC alloantibody is an important finding which may impact the HSCT process. Antibodies that developed during prior pregnancy or pre-HSCT blood product transfusion, specifically in patients with sickle cell disease, myelodysplastic syndrome or aplastic anemia, may be encountered; however, extended RBC antigen matching for blood transfusion may reduce alloimmunization to RBC antigens.^7,13,14^ RBC antibodies should be honored for all subsequent transfusions, and practitioners may choose to desensitize patients via plasma exchange, rituximab, daratumumab, or other methods, if antibodies are directed against donor RBC antigens.^14,15^ While data are limited, evidence from patients with sickle cell disease demonstrates that the presence of an RBC alloantibody contributes to increased transfusion burden, even in the setting of matched RBC transfusions.^16^ In a study of patients with RBC alloantibodies undergoing myeloablative HSCT, the transfusion burden was not increased, suggesting that clearance of recipient immune cells may reduce RBC transfusion requirements.^16,17^ Likewise, transplantation can lead to disappearance of alloantibodies present pre-transplant, further supporting this assertion.^18^ Ultimately, an RBC alloantibody directed against donor RBC antigens is not a contraindication to HSCT, although these antibodies may mediate hemolysis or prolonged reticulocytopenia.^19^ While the risk of alloimmunization during the engraftment period is low, this phenomenon does occur.^14,19,20^ Thus, patients will still require routine pre-transfusion testing for development or recrudescence of antibodies.

In RhD-mismatched HSCT, the recipient may infrequently develop anti-D alloantibodies, though these do not typically significantly impact patient outcomes.^21,22^

## Red Blood Cell Transfusion

In patients undergoing HSCT, erythroid engraftment is anticipated to occur approximately 3-4 weeks post stem cell infusion, generally after PLT engraftment. Most patients require RBC transfusion in the interim.^1,8^ It is difficult to make broad statements about engraftment for all populations, as the underlying disease, conditioning regimen and graft source all significantly affect engraftment times; however, independence from RBC transfusion, defined as no RBC transfusion for 30 days, is generally used as an indicator of engraftment .^23^ Similarly, conversion to donor ABO type in ABO incompatible transplant can be used, though this tends to occur later.

The most recent Association for the Advancement of Blood and Biotherapies (AABB) RBC transfusion guidelines provide specific guidance for hematology and oncology patients with an RBC transfusion threshold of 7 g/dL (conditional, low certainty evidence).^4^ It should be noted that, in patients undergoing cardiac surgery, a threshold of 7.5 g/dL is recommended, and for those undergoing orthopedic surgery or with history of cardiovascular disease, a higher threshold of 8 g/dL may be considered.^4^ A 2023 randomized controlled trial of patients with myocardial infarction and a hemoglobin level of less than 10 g/dL compared a restrictive transfusion strategy (hemoglobin cutoff for transfusion, 7 or 8 g/dL) to a liberal transfusion strategy (hemoglobin cutoff, <10 g/dL).^24^ That trial found no statistically significant differences in the primary outcome (recurrent myocardial infarction and death) between the groups, and potential harms of either a restrictive or liberal transfusion strategy could not be excluded.^24^ Nevertheless, the evidence for a restrictive RBC transfusion strategy in HSCT has demonstrated similar patient outcomes to liberal transfusion strategies.^25,26^

Given the pre-transplant transfusion requirements for many acquired bone marrow failure conditions, such as myelodysplastic syndrome or aplastic anemia, unnecessary transfusion should be avoided in these populations given the burden of iron overload. Each milliliter of RBCs contains one milligram of additional iron, and the body has no dedicated form of iron excretion. Thus, many of these patients require management for iron overload, a condition associated with poor engraftment during HSCT. The use of erythropoiesis-stimulating agent (ESA) therapy may also be considered.^27,28^

## Platelet Transfusion

Nearly all patients will require PLT transfusion during the post-transplant period. PLT engraftment typically occurs after neutrophil engraftment, at approximately two weeks.^1,8^ It can be defined as a PLT count of 20,000/μL for 7 days without transfusion.^29^

Based on the current evidence and guidelines endorsed by AABB, the American Society of Clinical Oncology (ASCO), and the British Society of Haematology (BSH), prophylactic PLT transfusion is recommended for adult patients with therapy-induced hypoproliferative thrombocytopenia at PLT counts <10,000/µL to reduce the risk of spontaneous intracranial hemorrhage.^30–32^ In patients undergoing autologous HSCT, bleeding events were similar in those receiving prophylactic PLT transfusion at 10,000/μL compared to those receiving symptomatic PLT transfusion (i.e., those developing WHO Grade 2-4 bleeding). These findings demonstrated that patients without evidence of bleeding may not require prophylactic platelet transfusion, and administering symptomatic platelet transfusion in this particular population may be safe, and significantly reduces platelet transfusion frequency.^5^ As such, adult patients who undergo autologous HSCT at experienced centers may receive platelet transfusion at the first sign of bleeding rather than prophylactically. In addition to this “symptomatic transfusion” approach, the impact of blood shortages in contemporary practice may necessitate lower thresholds such as 5,000/μL at some sites, which is supported by results from the Optimal Platelet Dose Strategy for Management of Thrombocytopenia (PLADO) trial, wherein the risk of bleeding did not change once the PLT count was >5,000/µL.^33^ However, this practice may not be evidence-based in all populations, and further investigation is necessary to establish if certain patient populations can tolerate lower counts without catastrophic bleeding.^34^

In patients with additional bleeding risk factors (e.g., fever, infection post-HSCT), the threshold for prophylactic platelet transfusion is typically increased. In this context, despite the low-quality data, the BSH guidelines recommend considering increasing the threshold for prophylactic platelet transfusion to between 10,000 and 20,000/ µL in patients with these risk factors for bleeding.^32,35^ While the ASCO guidelines do not provide a specific threshold, they also suggest that it should be higher in patients with additional bleeding risk factors.^31^

The recommended threshold at which PLT transfusion should be performed prior to procedures varies among society guidelines and institutional practices, with limited evidence from randomized trials to guide practice. For procedures such as central venous catheter (CVC) placement, AABB, ASCO, and BSH recommend prophylactic PLT transfusion for patients with PLT counts <20,000/µL. However, a recent randomized controlled, non-inferiority trial in patients with thrombocytopenia (10,000/uL to 50,000/uL) compared transfusion of one PLT unit to no transfusion prior to CVC placement, and found withholding PLT transfusion did not meet the predefined non-inferiority, and resulted in more CVC-related events.^36^ Nevertheless, this trial did not stratify patients by PLT count between 10,000/uL and 50,000/uL, and, therefore, a more nuanced transfusion threshold cannot be ascertained from these data.

Similarly, there is no definitive evidence to support a particular PLT threshold prior to more invasive procedures such as lumbar puncture (LP) or epidural anesthesia. AABB suggests prophylactic PLT transfusion for patients undergoing LP with a PLT count <50,000/µL, while the BSH recommends a threshold of <40,000/µL.^30,32,37^ This lower threshold is supported by a recent single-center study that compared PLT thresholds of 50,000/µL and 40,000/µL for patients undergoing LP, and found that patients in the 40,000/µL group received significantly fewer PLT transfusions, without an increased risk of complications; however, there was an increased incidence of traumatic taps (>10 RBCs/µL in cerebrospinal fluid), though the implication of this finding is unclear.^38^ One caveat that should be considered in these scenarios is the experience of the individual performing the procedure.

For most other invasive non-neuraxial procedures, ASCO recommends a PLT count of 40,000 to 50,000/µL, while AABB and BSH recommend a threshold of 50,000/µL.^30–32^ For central nervous system and posterior ocular procedures, a PLT threshold of 80,000 to 100,000/µL is typically used; however, there is only low-quality evidence available, with no explicit practice guidelines by the AABB or ASCO, though BSH does recommend a threshold of 100,000/µL.^32^

Most of the above recommendations are based on observations and studies in adult patients; thus, it is unclear whether these same thresholds can be extrapolated to pediatric oncology patients. The ASCO guidelines, many of which have been endorsed by the Children’s Oncology Group (COG), recommend a threshold of <10,000/µL for prophylactic PLT transfusion in pediatric patients undergoing allogeneic HSCT similar to adult patients.^39^ Like for adult patients, they also suggest that transfusion at higher levels may be considered in a subset of children, such as those with hemorrhage, fever, hyperleukocytosis, rapid PLT count decrease, coagulation abnormalities, or for outpatients who live at significant distances from treatment centers. However, they make no specific recommendations for these levels, and there are little trial data to support this. COG also endorses the ASCO guidelines regarding a PLT transfusion threshold of 40,000 to 50,000/µL prior to major non-neuraxial procedures, and <20,000/µL for bone marrow aspiration in pediatric patients.

PLT refractoriness, defined by suboptimal increment after transfusion, is an important clinical challenge frequently encountered during the peri-transplant period.^40,41^ While the majority of refractoriness is due to non-immune or non-antibody-mediated causes such as splenomegaly or fever, antibodies directed against HLA class I antigens or human platelet antigens (HPAs) may lead to increased clearance of transfused PLTs.^4^ Specifically, HLA antibodies can develop in response to pregnancy, transplantation, or transfusion, and are seen in up to half of patients undergoing HSCT.^3^ Leukoreduction reduces HLA sensitization from blood transfusion. Cardillo and colleagues demonstrated decreased HLA-antibody formation with the use of ABO-matched PLT transfusion.^41,42^ As donor-specific HLA antibodies can lead to poor engraftment and increased incidence of graft failure, patients with extensive HLA sensitization will require coordination between the clinical team, HLA laboratory, blood bank, and blood supplier to ensure safe peri-HSCT PLT transfusion.^3,40,43^ To that end, directed blood donations from family members prior to transplantation should be avoided to prevent alloimmunization to minor HLA antigens that could subsequently reduce the pool of the donors most likely to be HLA-compatible (i.e., family members).

As it is difficult to predict who will form HLA antibodies, and infeasible to prophylactically provide HLA-matched PLT transfusions, practitioners must be equipped to support these patients as best as possible once antibodies are present. PLT transfusion may be HLA-matched for the recipient or, more commonly, the recipient may receive serologically crossmatched units or PLTs lacking the HLA antigens against which they have developed antibodies.^40^ Desensitization with immunosuppressants to theoretically reduce the number of antibody-producing cells, or temporarily decreasing the antibody burden via plasma exchange, may be considered if a patient has donor-specific antibodies; however, patients may also develop anti-HLA antibodies after transplant, precipitating PLT refractoriness.^3,44^ Post-transplant immune thrombocytopenia can be challenging to manage, though one recent case report demonstrated efficacy in improving PLT counts utilizing daratumumab.^44^ Therefore, B-cell depletion therapy may be a promising therapeutic modality for ameliorating PLT transfusion refractoriness and warrants further investigation.

Finally, with regard to selection of RhD matching for PLT products in these patients, a survey by Poston et al demonstrated that the practice is variable and often inventory-dependent.^45^ PLTs do not express the RhD antigen, but PLT products do contain minimal amounts of RBCs or RBC fragments, hence the theoretical risk of RhD alloimmunization.^46^ Whole blood derived pooled PLTs have a higher RBC content relative to apheresis PLTs (~0.3 mL versus ~0.01 mL) and portend a greater risk of RhD alloimmunization; nevertheless, this risk remains low.^46^ One vial of Rh immune globulin will effectively provide prophylaxis against RhD alloimmunization following multiple units of apheresis-derived or whole blood-derived RhD+ PLT, and may be considered for patients with childbearing potential.^46^

## Granulocyte Transfusion

The use of granulocyte transfusion in patients undergoing HSCT is controversial. Neutrophils and white blood cells (WBCs) should engraft quickly, within the first week, and engraftment is defined by an absolute neutrophil count (ANC) greater than 500/µL.^1,47^ While granulocyte transfusion may be considered in patients with refractory bacterial or fungal infection in the setting of severe neutropenia (ANC <500/µL) with expected marrow recovery, the largest randomized control trial to date showed no clear benefit.^48^ However, the study was underpowered due to poor enrollment, and a significant effect in high-concentration dosing was observed; as such, there is a theoretical benefit in smaller adult or pediatric patients.^48^

Because they contain large amounts of donor RBCs, granulocyte units should be ABO-compatible with the recipient’s plasma to avoid hemolytic transfusion reactions.^49^ CMV matching should also be considered because granulocytes must not be leukoreduced, which can also lead to HLA sensitization.^49^ In addition, the product should also be irradiated to prevent transfusion associated-graft versus host disease (TA-GVHD).^49^ Products must be transfused within 24 hours, and a waiver must be signed, as infectious disease testing cannot be completed prior to release.^49^ Of note, granulocyte infusions are associated with fever and can precipitate more severe reactions such as transfusion-related acute lung injury.

Once granulocyte transfusion therapy is initiated, repeat infusions are generally prescribed daily until the infection is resolved, the patient defervesces, the ANC returns to greater than 500/ µL, or the practitioner elects to cease therapy.^49^ Blood centers typically recruit apheresis PLT donors with recent negative infectious disease testing for granulocyte donation. As large numbers of granulocytes is desired, mobilizing donors with both granulocyte-colony stimulating factor (G-CSF) and corticosteroids should be considered to optimize collections, although many blood centers mobilize with corticosteroids only.^50^ Given the challenges associated with obtaining apheresis granulocytes, there is some interest in utilizing buffy coat pooled granulocytes, though this practice is uncommon in the United States (US). Similarly, despite the potential risks, due to ongoing shortages and issues with efficacy, some sites have opted to forego ABO matching or irradiation to improve availability or white cell viability.^51,52^

## Plasma and Cryoprecipitate Transfusion

While a minority of patients have liver disease prior to HSCT, hepatic dysfunction may be exacerbated during the post-transplant period, often related to drug toxicity and/or graft-versus-host disease (GVHD).^53^ This may lead to perturbations in coagulation laboratory values; however, these abnormal laboratory results do not necessarily associate with a hemorrhagic diathesis, as hepatic synthesis of both natural pro- and anti-coagulants is altered. Moreover, the inflammatory state, with increased production of procoagulant factors (e.g., factor VIII and von Willebrand factor), in conjunction with disease- and transplant-related factors (e.g., GVHD), may predispose to thrombosis.^54^ As such, single unit plasma transfusion remains contraindicated, and will not correct minor prolongations in the international normalized ratio (INR).^55^ However, cryoprecipitate or fibrinogen concentrate may be indicated in the setting of severe hypofibrinogenemia, although there is minimal evidence to inform practice regarding prophylactic fibrinogen thresholds in the absence of bleeding.

## Product Modifications

Given the immune changes surrounding pre-HSCT chemotherapy, special considerations should be reviewed regarding the safety of blood products. As such, special product modifications are required for this population.

Foremost amongst these product modifications is leukoreduction. The majority of blood products transfused in the USA are leukoreduced, and this is crucial for the HSCT patient population to prevent CMV transmission.^49^ Comparison of CMV-seronegative blood with leukoreduced blood demonstrated equivalent outcomes in transfusion-transmitted CMV infection.^56^ Accordingly, leukoreduction is increasingly accepted as equivalent to CMV-safe, with blood suppliers having limited, if any, CMV-tested products. In addition to protecting against CMV transmission, as described earlier, leukoreduction has the additional benefit of reducing HLA sensitization.^57^ Furthermore, pre-storage leukoreduction decreases the incidence of febrile non-hemolytic transfusion reactions, secondary to a reduction in cytokines produced by the fewer WBCs stored and transfused in the leukoreduced product.^57^

In addition to leukoreduction, all cellular blood products should be irradiated to prevent TA-GVHD.^49^ Although some authors have questioned whether irradiation is necessary for patients undergoing reduced intensity conditioned HSCT, there is no convincing evidence that these patients should not receive irradiated products, given the potential severity of TA-GVHD.^58,59^ Nevertheless, irradiation does adversely affect RBC products, including reducing the outdate to 28 days or the original outdate, whichever is sooner.^49^ Likewise, irradiation induces RBC membrane damage and precipitates potassium efflux from RBCs, thereby increasing the potassium content of the product’s extracellular fluid.^49^ This can be of some concern for pediatric patients, patients with diminished renal function, and those with cardiac conditions that make them sensitive to electrolyte disturbances. Finally, there is no consensus on the length of time a patient must receive irradiated blood products; however, the BSH recommends that all patients undergoing HSCT, irrespective of the underlying diagnosis, receive irradiated cellular blood components, from initiation of conditioning chemo/radiotherapy until three months post-transplant, or six months if total body irradiation is used in conditioning. Notably, some patients may require indefinite irradiation based on their conditioning regimen, disease, or previous therapy (e.g., Hodgkin lymphoma or prior purine analog treatment).^59^

Recent advances have led to the development of pathogen reduction technology (PRT). In the US, the only Food and Drug Administration (FDA)-approved method uses the addition of a psoralen compound during manufacturing, with subsequent exposure to ultraviolet (UV) light, thereby inactivating microorganisms due to the resultant nucleic acid damage.^60^ Other methodologies utilizing riboflavin and UV light or UV light alone are available in other regions. Unlike non-pathogen reduced PLTs that require multiple days of testing prior to release from the donor center, pathogen-reduced (PR) PLTs (PRPs) can be immediately transferred to hospital transfusion services, reduce the risk of CMV transmission, and are considered irradiation-equivalent for mitigating the risk of TA-GVHD. As PRPs require no additional modifications or testing to satisfy US FDA irradiation or bacterial contamination testing requirements, this modification makes these units attractive options for blood banks.^61^ However, PRPs are not without disadvantages, and in a 2020 survey of Transfusion Medicine directors, PRPs were considered less favorable compared to large-volume delayed sampling (LVDS) PLT products, due to their increased cost, lower corrected count increments (CCIs), and debated increase in HLA alloimmunization.^62–65^ Moreover, PRT does not preclude the possibility of septic transfusion reactions, as any organism introduced into the product following the PR process, during manufacturing or in the hospital, will not be inactivated. Nevertheless, PRPs and PR cryoprecipitate are currently available in the US, and PR RBCs are currently undergoing clinical trials.^60,66^ Notably, despite the less optimal PLT increments associated with PRPs, many blood suppliers are moving toward increasing their PRP inventories, given safety concerns (e.g., bacterial contamination) associated with traditional PLT products.^60^

Although plasma transfusions are infrequent among patients undergoing HSCT, solvent-detergent treated pooled plasma is also available.^67^ Though originally developed as a PR product, this specialized plasma product may be most advantageous in patients with recurrent allergic reactions to standard plasma.^68^ These novel product modifications highlight the blood industry’s further movement towards safer products, and PRT RBC products are currently being developed. Moving forward, HSCT centers must weigh the safety benefits of these products against the increased cost and other potential disadvantages.

## ABO-Incompatible Transplantation

Although HLA-matching requirements exist for HSCT, the ABO barrier can be safely crossed, and approximately half of transplants are ABO incompatible.^69,70^ Outcomes are heterogenous across groups, but overall the clinical course is similar for these patients.^70,71^

There are three types of ABO-incompatibility: major incompatibility (donor RBCs are incompatible with the recipient’s plasma containing isohemagglutinins); minor incompatibility (donor plasma containing isohemagglutinins and immune cells are incompatible with recipient RBCs); and bi-directional incompatibility (both donor and recipient have incompatible RBCs and plasma with isohemagglutinins and immune cells) (**Table 1**). Patients should regularly undergo scheduled ABO typing throughout HSCT, and notes should be made in the blood bank laboratory information system. Similarly, a policy detailing when to transition an individual’s ABO type to the donor’s type should be in place.

**Table 1.** Types of ABO incompatible hematopoietic stem cell transplant

ABO-Incompatibility in HSCT	Donor → Recipient
Major (incompatible donor RBCs)	A → O B → O AB → O AB → A AB → B
Minor (incompatible donor isohemagglutinins and immune system)	O → A O → A O → AB
Bidirectional (incompatible donor RBCs, isohemagglutinins, and immune system)	A → B B → A

The table categorizes ABO incompatible hematopoietic stem cell transplant by donor and recipient ABO typing.

During the HSCT process, the donor and recipient’s blood type and immune system must be considered, with products compatible with both parties used preferentially (**Table 2**).

**Table 2.** Preferred blood products for patients undergoing ABO incompatible hematopoietic stem cell transplant

Recipient	Donor	Type of Mismatch	Transplantation to RBC engraftment					Engraftment established				
			RBC	Platelets		Plasma		RBC	Platelets		Plasma	
				1^st^ Choice	2^nd^ Choice	1^st^ Choice	2^nd^ Choice		1^st^ Choice	2^nd^ Choice	1^st^ Choice	2^nd^ Choice
O	A	Major	O	A	AB, B, O	A	AB	A	A	AB, B, O	A	AB
O	B	Major	O	B	AB, A, O	B	AB	B	B	AB, A, O	B	AB
O	AB	Major	O	AB	A, B, O	AB	NA	AB	AB	A, B, O	AB	NA
A	O	Minor	O	A	AB, B, O	A	AB	O	A	AB, B, O	A	AB
A	B	Bidirectional	O	AB	B, A, O	AB	NA	B	AB	B, A, O	AB	NA
A	AB	Major	A	AB	A, B, O	AB	NA	AB	AB	A, B, O	AB	NA
B	O	Minor	O	B	AB, A, O	B	AB	O	B	AB, A, O	B	AB
B	A	Bidirectional	O	AB	O, A, B	AB	NA	A	AB	A, B, O	AB	NA
B	AB	Major	B	AB	B, A, O	AB	NA	AB	AB	B, A, O	AB	NA
AB	O	Minor	O	AB	A, B, O	AB	NA	O	AB	A, B, O	AB	NA
AB	A	Minor	A	AB	A, B, O	AB	NA	A	AB	A, B, O	AB	NA
AB	B	Minor	B	AB	B, A, O	AB	NA	B	AB	B, A, O	AB	NA

This table lists the preferred product ABO typing for patients undergoing ABO incompatible hematopoietic stem cell transplant. The goal is to have compatible cells and plasma with both the donor and recipient, if at all possible.

## Major ABO-Incompatibility

The risk of acute hemolysis due to the RBC content of an HSCT graft is important. Products are typically centrifuged to deplete donor RBCs before cryopreservation or infusion.^71^ The freeze-thaw process may also impact the amount of RBCs in the graft.^71^ Some authors have described utilizing plasma from blood donors who have an active secretor gene (i.e., individuals whose plasma contains circulating ABO antigens) to adsorb circulating isohemagglutinins in the recipient and similarly have attempted adsorptions using RBCs with the donor’s ABO type, though this practice is controversial.^72^

The greatest long-term concern for major ABO-incompatible HSCTs is delayed RBC engraftment, including pure red cell aplasia (PRCA), which has historically occurred in 8-26% of these patients.^71–73^ PRCA occurs when ABO antigens expressed on erythroid precursors are targeted by antibodies being produced by long-lived recipient plasma cells. These manifestations are characterized by ongoing anemia and reticulocytopenia.^71^ Immune interventions, including therapeutic plasma exchange, rituximab, and daratumumab, are among various immunomodulatory treatments for this complication.^71,74^ PRCA patients have increased RBC transfusion and iron burdens and may ultimately require additional HSCT.

## Minor ABO-Incompatibility

Minor ABO-incompatibility may present with acute hemolysis upon graft infusion. This can depend on the amount of plasma in the graft, which is often reduced before infusion. Given the large number of RBCs and tissue surfaces that express ABO antigens in the recipient, significant acute hemolysis is uncommon.

The most important manifestation of minor ABO-incompatible HSCT is passenger lymphocyte syndrome (PLS). In this condition, donor plasma cells contained within the transplanted graft will elaborate increasing levels of isohemagglutinins directed at recipient RBCs, which can lead to hemolysis at days five to 15 post-HSCT.^71^ This generally presents with a positive direct antiglobulin test (DAT) and is self-limited.^71^ It is important to note that, albeit less frequent, PLS can occur with non-ABO RBC antigen incompatibility (e.g., RhD, K), as well.

An additional point to consider for patients undergoing minor ABO-incompatible HSCT is that these individuals tend not to develop anti-recipient isohemagglutinins, which can lead to blood typing issues.^75^

## Bidirectional ABO-Incompatibility

Bidirectional ABO-incompatibility is associated with the same risks as both minor and major incompatible HSCT. To that end, it is the least commonly performed of the three incompatible transplants.^70^ These patients may also demonstrate inventory challenges if group ABO PLT products are necessary.

## Post-Transplant Endothelial Disorders

It has been recognized that endothelial injury occurs after HSCT leading to significant morbidity and mortality.^76^ This dysfunction is multi-factorial, and is a result of both the transplant as well as the conditioning regimen. Certain disorders requiring transfusion intervention are detailed below.

Post-transplant thrombotic microangiopathy (TMA) occurs in approximately 10% of patients and is associated with significant mortality. These patients can develop increased transfusion burden due to the consumption of PLTs in microthrombi and the development of hemolytic anemia.^77^ Historically, therapeutic plasma exchange has been employed, though contemporary evidence suggests this process is complement-mediated, and complement inhibitors (e.g., eculizumab) are now first-line therapy.^78^ Given the propensity of patients with post-transplant TMA to bleed, the current American Society for Apheresis (ASFA) guidelines recommend the use of plasma or a combination of albumin and plasma (with plasma used after albumin) as an exchange fluid if plasma exchange is employed.^79^

Hepatic sinusoidal obstruction syndrome (SOS)/veno-occlusive disease (VOD) is associated with endothelial damage and sloughing with intraparenchymal hemorrhage within the liver post-HSCT.^80^ Importantly, this condition also associates with increased transfusion burden, with new-onset PLT refractoriness representing a diagnostic criteria for SOS/VOD.^80,81^ Notably, this condition may require therapeutic anticoagulation, thus necessitating higher PLT transfusion thresholds, thereby creating additional challenges for the transfusion medicine service in supporting PLT-refractory patients. Additional therapeutic modalities, such as antithrombin replacement are beyond the scope of this review, but may be necessary in patients with reduced hepatic synthetic function requiring systemic anticoagulation.

## Post-Transplant Autoimmune Cytopenias

While infrequent, autoimmune hemolytic anemia, immune thrombocytopenia, and autoimmune neutropenia can occur post-HSCT.^72,82^ Although it is difficult to predict which patients will develop these manifestations, there is a trend toward autoimmune manifestations in patients with non-malignant indications for HSCT and those developing GVHD. Autoimmune hemolytic anemia has been described to occur most frequently. It is associated with multiple HSCT and CMV activity, though studies are mixed on whether naivety or reactivation is of greater risk.^72,82–84^ Patients will generally require both immunomodulatory support and ongoing blood product transfusion.

## Additional CD34 cell administration for poor graft function

While not performed at all centers, practitioners may consider providing additional CD34 selected stem cells for patients with cytopenia after engraftment, described as poor graft function.^85,86^ Also referred to as a ‘stem cell boost,’ this infusion helps buoy marrow production, and meta-analysis data demonstrate high-level complete response rates (CRR), 72%, defined as subsequent transfusion independence.^86^ These findings were replicated in a large single-center retrospective study, with 72.5% achieving CRR.^85^ Risks include high rates of acute GVHD and residual risk for mortality.

## Conclusion

As the number of patients undergoing HSCT increases, blood transfusion remains a pillar of peri-transplant therapy. All the while, developments in transfusion guidelines and immunomodulatory medications continue to impact the transfusion methods employed for these patients. Transplantation and transfusion medicine physicians should be familiar with contemporary HSCT practices, as well as anticipated transfusion needs for this population, to ensure optimal outcomes.

### Conflicts of interest

All authors declare no conflicts of interest.
